# Genetic polymorphisms of inflammatory and bone metabolism related proteins in a population with dental implants of the Basque Country. A case-control study

**DOI:** 10.1186/s12903-024-04319-1

**Published:** 2024-06-05

**Authors:** Irene Lafuente-Ibáñez-de-Mendoza, Xabier Marichalar-Mendia, Amaia Setién-Olarra, Ana María García-de-la-Fuente, Rafael Martínez-Conde-Llamosas, José Manuel Aguirre-Urizar

**Affiliations:** 1 Research Group: GIU21/042, University of the Basque Country (UPV/EHU), Leioa, Spain; 2 Department of Stomatology, University of the Basque Country (UPV/EHU), Barrio Sarriena s/n, Leioa, 48940 Spain; 3 Department of Nursery I, Barrio Sarriena s/n, Leioa, 48940 Bizkaia Spain; 4 Biobizkaia Health Research Institute, Barakaldo, Spain

**Keywords:** Peri-implantitis, Single nucleotide polymorphisms, Case-control studies, Inflammation, Bone metabolism

## Abstract

**Background:**

Peri-implantitis (PI) is a frequent inflammatory disorder characterised by progressive loss of the supporting bone. Not all patients with recognised risk factors develop PI. The aim of this study is to evaluate the presence of single nucleotide polymorphisms (SNP) of inflammatory and bone metabolism related proteins in a population treated with dental implants from the Basque Country (Spain).

**Methods:**

We included 80 patients with diagnosis of PI and 81 patients without PI, 91 women and 70 men, with a mean age of 60.90 years. SNPs of BMP-4, BRINP3, CD14, FGF-3, FGF-10, GBP-1, IL-1α, IL-1β, IL-10, LTF, OPG and RANKL proteins were selected. We performed a univariate and bivariate analysis using IBM SPSS® v.28 statistical software.

**Results:**

Presence of SNPs GBP1 rs7911 (*p* = 0.041) and BRINP3 rs1935881 (*p* = 0.012) was significantly more common in patients with PI. Patients with PI who smoked (> 10 cig/day) showed a higher presence of OPG rs2073617 SNP (*p* = 0.034). Also, BMP-4 rs17563 (*p* = 0.018) and FGF-3 rs1893047 (*p* = 0.014) SNPs were more frequent in patients with PI and Type II diabetes mellitus.

**Conclusions:**

Our findings suggest that PI could be favoured by an alteration in the osseointegration of dental implants, based on an abnormal immunological response to peri-implant infection in patients from the Basque Country (Spain).

## Introduction

The first cases of peri-implantitis (PI) were described as inflammatory reactions leading to bone loss around a functioning dental implant [1]. Currently, PI is considered a peri-implant disease characterised by inflammation of the peri-implant mucosa and progressive loss of the supporting bone [2]. It is an infectious disease with a multifactorial nature, affecting up to 50% of patients with dental implants [2].

The main risk factors for developing PI are: (1) previous clinical history of periodontitis, and (2) poor plaque control and/or maintenance therapy [3]. However, other factors such as smoking and Type II diabetes mellitus may also be related [2]. Patients with periodontal background show higher probing depth (> 6 mm) and marginal bone loss (> 3 mm) numbers than those without periodontitis; as well as a higher rate of conversion from peri-implant mucositis to peri-implantitis (31%) (Roccuzzo et al., 2010; Roccuzzo et al., 2012). Furthermore, presence of active periodontitis during dental implant therapy is a 4- to 7-fold risk factor for developing PI (Máximo et al., 2008; Koldsland et al., 2010; Koldsland et al., 2011; Casado et al., 2013; Renvert et al., 2014; de Araujo Nobre et al., 2015; Daubert et al., 2015; Dalago et al., 2017). Active control of dental implants allows assessment of bacterial plaque accumulation, as well as changes in clinical data. Thus, maintenance therapy is key in the management of this disease, for both patient monitoring and primary prevention (Aguirre-Zorzano et al., 2015). Up to a 14-fold increased risk of PI has been reported in patients not attending a supervised treatment, with an incidence of PI of 44% compared to 18% in patients following active control therapy (Ferreira et al., 2006; Rokn et al., 2017).

Given that not all individuals with this background develop PI [2, 4], a facilitating genetic susceptibility could explain the development this disorder in some individuals [5].

Genetic polymorphisms are variations at a particular point in the DNA sequence, which occur in more than 1% of the population [6]. Single nucleotide polymorphisms (SNP) are the most frequent genetic polymorphisms [6]. The detection of these alterations can be used to identify the genes and proteins involved in a specific disease; thus, their study allows population-based genetic predisposition analysis [7].

The most studied SNPs in the context of PI belong to inflammatory interleukins IL-1β, IL-1α, and IL-10 [8–11] and proteins related to bone metabolism BMP-4, BRINP3, CD14, FGF, LTF, OPG and RANKL [12–15]. Since PI is an inflammation-based disorder that causes bone loss, these mutated genes could trigger an abnormal inflammatory response and/or a reduced peri-implant osseointegration, which lead to peri-implantitis. These inflammatory and bone-related molecules may work as effective diagnostic tools or personalized treatment approaches as potential biomarkers of PI for individuals at higher genetic risk.

Sadly, so far, only the C/C genotype of IL-1β (-511) has been statistically linked to PI [16]. Although a highly suggestive avenue of study, the true implication of genetic susceptibility and the existence of specific SNPs in relation to the development of PI has not yet been demonstrated. We believe the reason behind these poor results is due to the methodology of the studies being heterogeneous, differences in diagnostic evaluation, population heterogeneity, and exclusion of some important parameters like history of periodontitis and tobacco consumption.

In this regard, there is little research on the association between PI and single nucleotide polymorphisms in the Spanish population (García-Delaney et al., 2007), which makes necessary to perform larger studies, with well-selected samples and an updated and specific methodological design. The objective of this study is, therefore, to perform a genetic study in a population from the Basque Country (Spain) treated with dental implants, with and without PI, to determine the SNP profile of inflammatory and bone metabolism related proteins.

## Methods

### Study design and participants

A retrospective study was carried out at the Periodontology and Osseointegration Unit of the Dental Clinic Service of the University of the Basque Country and the Centro Odontológico Médico Quirúrgico. Because of the unpredictable nature of PI and its onset, the methodology of case-control studies ensures sample size reach, as PI is sometimes has a long latency. Accepting an alpha risk of 0.05 and a beta risk of 0.2 in a two-sided test, 78 cases and 78 controls were needed to recognize as statistically significant odds ratios greater than or equal to 3.

This project matched STROBE guidelines and was approved by the University of the Basque Country/EHU Research Ethics Committee (CEISH: M10/2016/057, CEIAB/2016/180). The data extracted from this study were physically stored in an anonymised form with a code assigned to each patient in the computer of the head of research. A back-up copy in a hard disk/usb format was also made.

Patients were selected on their dental visit, upon those who underwent implant maintenance therapy. We approached 161 individuals in total: 80 patients with PI (case group, CAG) and 81 patients without PI (control group, COG). These corresponded to 91 women (56.50%) and 70 men (43.50%), whose mean age was 60.90 *±* 10.22 (range: 31–86). All participants signed an informed consent form at the baseline before participating in the study. Two experienced and calibrated specialists in oral surgery and periodontology, who followed the same maintenance therapy for patients with dental implants and were blinded to the case/control of patients made the clinical and radiographic assessment (AMG, RMC) in order to enhance transparency.

Case participants were diagnosed with PI on at least one dental implant [3]: (1) Evidence of visual inflammatory changes in the peri-implant soft tissues combined with bleeding on probing and/or suppuration, (2) Increasing probing pocket depths as compared to measurements obtained at placement of the supra-structure, (3) Progressive bone loss in relation to the radiographic bone level assessment at 1 year following the delivery of the implant-supported prosthetics reconstruction, and (4) In the absence of initial radiographs and probing depths, radiographic evidence of bone level ≥ 3 mm and/or probing depths ≥ 6 mm in conjunction with profuse bleeding represents peri-implantitis. Control participants only had healthy implants without PI.

Inclusion criteria for patients were: (1) being over 18 years old, (2) having at least one functional dental implant for a minimum of 1 year, and (3) being enrolled in a supportive program therapy protocol [17]. Exclusion criteria were: (1) having received periodontal-tissue healing, antibiotics and/or bone metabolism related drugs in the last 6 months prior to genetic sampling, (2) having cement-retained implant-supported dental restorations, and (3) having been surgically treated for PI in the last 6 months prior to genetic sampling.

### Clinical and radiographic analysis

We gathered the following clinical features of the participants: age (years), gender (female, male), follow-up time (months), smoking habit (more or less than 10 cigarettes per day), alcohol consumption (number of alcoholic units per week) and systemic diseases. Individuals who have quitted smoking for more than 10 years were considered former smokers. History of periodontitis (aggressive, chronic) [18] was also collected.

We registered the following dental implant data: number, location, probing depth (PD), bleeding on probing (BOP), suppuration on probing, and marginal bone loss (MBL) (through periapical intraoral radiographs with parallel technique at the time of genetic sampling). In case of more than one dental implant with PI, the implant with the highest PD and MBL was considered for the analyses.

If any clinical and/or radiographic data was missing at the baseline of the study, this information was obtained at future monthly maintenance follow-ups.

### DNA isolation and genotyping analysis

Samples for the genetic analysis were taken by vigorous scraping of the buccal mucosa with a Rovers Orcellex® nylon brush (Lekstraat, The Netherlands), which was later placed in 2.5 ml of sterile miliQ water and stored at -20 °C [19].

Sample thawing was performed in a 37ºC bath with agitation. We used a standard Qiagen® kit (Redwood City, USA) for the DNA extraction, taking 1.5mL of the oral scraping sample. DNA quantification was made with a ThermoScientific NanoDrop™ spectrophotometer (Massachusetts, USA) (260/280 or 260/230 ratios). We obtained the DNA via fluorometric analysis, at concentrations 2.5–250 ng/uL.

For the genotyping analysis, specific target amplifications of the genomic regions of the SNPs were performed, then 96.96 JUNO™ chips were loaded (IFC - integrated fluidic circuits - controller), and finally allele-specific RT-PCR for each SNP and sample were made.

For the selection of the specifics SNPs, a systematic review of the literature was carried out [5] as well as an in-depth study on the presence of SNPs in the Caucasian population of the Basque County. only those related to the inflammatory response and bone metabolism made the final cut. Finally, we analysed the following polymorphisms with the Fluidigm SNP Genotyping Analysis® Software (San Francisco, USA): BMP-4 (rs17563), BRINP3 (rs1935881), CD14 (rs2569190), FGF-3 (rs1893047), FGF10 (rs900379), GBP-1 (rs7911), IL-1α (rs1800795), IL-1β (rs16944), IL-10 (rs1800896), LTF (rs1126477), OPG (rs2073617), RANKL (rs9533156). Biomark Data Collection™ software (San Francisco, USA) was used for data collection.

Two blinded biologist to the case/control status of the individuals (XMM, ASO), made all the genetic study process.

### Statistical analysis

First, we performed a univariate description with frequencies and percentages (qualitative variables), as well as means, standard deviations and ranges (quantitative variables). Secondly, a bivariate analysis was implemented: Chi-square test (categorical variables), Student’s t-test (quantitative and categorical variables) or Mann Whitney U-test (quantitative with abnormal distribution and categorical variable). For multivariate analysis, a logistic regression model was used. It was considered statistically significant when *p* < 0.05. We used IBM SPSS® v.28 statistical software.

In addition, Hardy-Weinberg Equilibrium (HWE) analysis was stablished using the Chi-square test. Hardy-Weinberg equilibrium describes a state in which allele and genotypic frequencies in a population remain constant from one generation to the next, under certain ideal conditions, such as the absence of natural selection, migration, mutation and genetic drift. None of the polymorphisms showed an imbalance.

A blinded biostatistician, who was blind to the case/control nature of the participants (XMM) carried the statistical analysis.

## Results

### Patient population

The main clinical data of the study groups are shown in Table 1. Clinical follow-up of the patients was longer in the CAG (mean: 7.45 *±* 3.19 months; range: 2–13) (*p* < 0.01). The mean time between implant placement and PI diagnosis was 4.61 *±* 2.5 years (range: 1–10).

**Table 1:** Epidemiological and clinical data of the patients included in the study

DATA	CAG (n: 80)	COG (n: 80)	Total (n: 161)	*p*
Gender: n (%)				0.58
• Female	43 (53.80)	48 (59.30)	91 (56.50)	
• Male	37 (46.30)	33 (40.70)	70 (43.50)	
Age (years)				0.374
• Mean ± SD	61.23 ± 8.92	60.70 ± 11.41	60.90 ± 10.22	
• Range	(37–77)	(31–86)	31–86	
Age at PI diagnosis (years)	-		-	-
• Mean ± SD		58.60 ± 8.90		
• Range		(32–76)		
Follow-up (years)				**< 0.01**
• Mean ± SD	7.45 ± 3.19	5.41 ± 3.14	6,43 ± 3,32	
• Range	(2–13)	(1–13)	(1–13)	
Tobacco consumption: n (%)				
• Non-smoker	22 (27.5)	40 (49.40)	62 (38.50)	**< 0.05**
• <10 cig/day	4 (5)	5 (6.20)	9 (5.60)	
• ≥10 cig/day	17 (21.4)	6 (7.40)	23 (14.20)	**0.009**
• Former smoker < 10 cig/day	2 (5.40)	3 (10)	5 (7.50)	0.65
• Former smoker ≥ 10 cig/day	35 (94.60)	27 (90)	62 (92.50)	
Medical history: n (%)				
• Arterial hypertension	22 (27.50)	25 (30.90)	47 (29.20)	0.22
• Hypercholesterolemia	12 (15)	13 (16)	25 (15.50)	0.854
• Depression	4 (5)	9 (11.10)	13 (8.10)	2.025
• Hypothyroidism	7 (8.80)	5 (6.20)	12 (7.50)	0.388
• Asthma	6 (7.50)	6 (6.20)	11 (6.80)	0.111
• Type II diabetes mellitus	4 (5)	6 (7.40)	10 (6.20)	0.388
• Cardiovascular disease	4 (5)	6 (7.60)	10 (6.20)	0.334
• Malignancy	4 (5)	5 (6.2)	9 (5.60)	0.105
• Hiatus hernia	3 (3.8)	5 (6.2)	8 (5)	0.194
History of periodontitis: n (%)				0.503
• Yes	34 (42.50)	29 (35.80)	63 (39.10)	
• No	46 (57.50)	52 (64.20)	98 (60.90)	

CAG: case group; COG: control group; PI: peri-implantitis; SD: standard deviation

Overall, 39.10% of participants (n: 63) had a history of periodontal disease, this number being higher in the CAG (42.50%) (*p* = 0.503). There were more former smokers in the COG (49.40%) (*p* < 0.05), and more heavy current smokers (> 10 cig/day) in the CAG (21.30%) (*p* = 0.009) (Table 1).

Arterial hypertension was the most frequent medical pathology in both study groups (29.20%), followed by hypercholesterolemia (15.50%), depression (8.10%), hypothyroidism (7.50%), bronchial asthma (6.80%), Type II diabetes mellitus (6.20%) and cardiovascular disease (6.20%) (Table 1).

### Clinical and radiographic outcomes

The 161 individuals included in the study had a total of 799 dental implants, out of which 60.20% were located in the mandible, 39.80% in the upper maxilla (*p* = 0.676), 72.23% in the posterior sector and 27.76% in the anterior sector (*p* = 0.713). Table 2 shows the clinical data of the 229 implants with PI (28.66%) and the 570 without PI.

**Table 2:** Data of the dental implants analysed in the study

DATA	Peri-implantitis (*n*: 229)	Peri-implant health (*n*: 570)	Total (*n*: 799)	*p*
Number by patient (n)				0.172
• Mean	5.23 ± 3.75	4.70 ± 3.22	4.96 ± 3.49	
• Range	(1–14)	(1–14)	(1–14)	
Location: n (%)				
• Anterior sector	68 (29.70)	167 (29.30)	229 (28.90)	0.713
• Posterior sector	161 (70.30)	403 (70.70)	564 (71.10)	
• Upper maxilla	87 (38)	221 (38.80)	318 (39.80)	0.676
• Mandible	142 (62)	349 (61.20)	481 (60.20)	
PD (mm)				**< 0.01**
• Mean ± SD	5.13 ± 1.25	1.72 ± 1.00	3.41 ± 1.96	
• Range	(2–9)	(0–3)	(0–9)	
MBL (mm)				**< 0.01**
• Mean ± SD	5.58 ± 1.13	2.42 ± 1.18	3.99 ± 2.05	
• Range	(3–10)	(0–4)	(0–10)	
BOP: n (%)				**< 0.01**
• Yes	129 (56.30)	31 (5.40)	160 (20)	
• No	100 (43.80)	539 (94.60)	639 (80)	
Suppuration: n (%)				**< 0.01**
• Yes	32 (14)	0 (0)	32 (4)	
• No	197 (86)	570 (100)	767 (96)	

BOP: bleeding on probing; MBL: marginal bone loss; PD: probing depth; SD: standard deviation

At the time of diagnosis, the implants with PI had a mean PD of 5.13 *±* 1.25 mm (range: 2–9) and a mean MBL of 5.58 *±* 1.13 mm (range: 3–10). Also, 56.30% (n: 129) PI cases had BOP and 14% showed signs of suppuration (n: 32). All these differences were statistically significant (*p* < 0.001) (Table 2).

### Genetic analysis

The results of the genetic analysis are displayed in Tables 3 and 4. All SNPs were consistent with HWE (Table 3). The overall comparative analysis showed that only GBP1 rs7911 (*p* = 0.041) and BRINP3 rs1935881 were significantly more common in patients with PI (*p* = 0.012) (Table 3), but none of them significantly increased the risk of developing PI (Table 5).

**Table 3:** Genetic data. Overall comparative analysis of the selected SNPs

Gen-SNP	Genotype	CAG	COG	*p*	HWE *p*
BMP-4 rs17563	CC	20 (27)	16 (20.3)	0.597	0.696
	CT	34 (45.9)	41 (51.9)		
	TT	20 (27)	22 (27.8)		
BRINP3 rs1935881	AA	41 (55.4)	26 (32.9)	**0.012**	0.073
	AG	25 (33.8)	45 (57)		
	GG	8 (10.8)	8 (10.1)		
CD14 rs2569190	AA	18 (24.3)	16 (20.3)	0.588	0.211
	AG	36 (48.6)	45 (57)		
	GG	20 (27)	18 (22.8)		
FGF-3 rs1893047	CC	17 (23)	18 (22.8)	0.688	0.928
	CT	32 (43.2)	39 (49.4)		
	TT	25 (33.8)	22 (27.8)		
FGF-10 rs900379	CC	11 (14.9)	8 (10.1)	0.641	0.833
	CT	31 (41.9)	33 (41.8)		
	TT	32 (43.2)	38 (48.1)		
GBP1 rs7911	CC	16 (21.6)	12 (15.2)	**0.041**	0.110
	CT	28 (37.8)	46 (58.2)		
	TT	30 (40.5)	21 (26.6)		
IL-1α rs1800795	CC	9 (12.3)	6 (7.7)	0.62	0.07
	CG	37 (50.7)	43 (55.1)		
	GG	27 (37)	29 (37.2)		
IL-1β rs16944	AA	7 (9.5)	6 (7.6)	0.795	0.401
	AG	37 (50)	37 (46.8)		
	GG	30 (40.5)	36 (45.6)		
IL-10 rs1800896	AA	23 (31.1)	33 (41.8)	0.389	0.340
	AG	37 (50)	33 (41.8)		
	GG	14 (18.9)	13 (16.5)		
LTF rs1126477	AA	5 (6.8)	3 (3.8)	0.409	0.820
	AG	27 (36.5)	23 (29.5)		
	GG	42 (56.8)	52 (66.7)		
OPG rs2073617	CC	11 (14.9)	18 (22.8)	0.448	0.470
	CT	36 (48.6)	36 (45.6)		
	TT	27 (36.5)	25 (31.6)		
RANKL rs9533156	CC	12 (16.2)	15 (19)	0.903	0.866
	CT	37 (50)	38 (48.1)		
	TT	25 (33.8)	26 (32.9)		

CAG: case group; COG: control group; HWE: Hardy-Weimberg Equilibrium; SNP: single nucleotide polymorphism

**Table 4:** Genetic data. Overall comparative analysis of SNPs according to PI risk factors

Gen-SNP	Study group	Genotype	Tobacco			*p*
			No	< 10 cig/day	*≥* 10 cig/day	
OPG rs2073617	COG	CC	8 (20.50)	1 (20)	2 (33.33)	**0.034**
		CG	19 (48.70)	4 (80)	4 (66.70)	
		GG	12 (30.80)	0 (0)	0(0)	
	CAG	CC	2 (100)	0 (0)	3 (25)	
		CG	7 (46.70)	2 (100)	9 (75)	
		GG	6 (40)	0 (0)	0 (0)	
**Gen-SNP**	**Study group**	**Genotype**	**Type II diabetes mellitus**			***p***
			**No**	**Yes**		
BMP-4 rs17563	COG	CC	15 (20.50)	1 (16.70)		**0.018**
		CT	37 (50.70)	4 (66.70)		
		TT	21 (28.80)	1 (16.70)		
	CAG	CC	13 (24.10)	3 (100)		
		CT	28 (51.90)	0 (0)		
		TT	13 (24.10)	3 (100)		
FGF-3 rs1893047	COG	AA	13 (17.80)	2 (33.33)		**0.014**
		AG	38 (52.10)	2 (33.33)		
		GG	22 (30.10)	2 (33.33)		
	CAG	AA	14 (25.90)	0 (0)		
		AG	28 (51.90)	0 (0)		
		GG	12 (22.20)	3 (100)		

CAG: case group; COG: control group; PI: peri-implantitis; SNP: single nucleotide polymorphism

**Table 5:** Binary logistic regression of the genetic data

Gen-SNP	Genotype	OR (IC95%)	*p*	Adjusted OR (IC95%)	*p*
GBP1 rs7911	CC	1	-	1	-
	CT	0,46 (0,189–1,105)	0,08	0,51 (0,20 − 1,28)	0,15
	TT	1,07 (0,421–2,725)	0,89	1,30 (0,49 − 3,47)	0,80
BRINP3 rs1935881	GG	1	-	1	-
	AG	0,56 (0,186–1,661)	0,29	0,48 (0,15 − 1,47)	0,20
	AA	1,58 (0,527–4,720)	0,42	1,34 (0,45 − 4,30)	0,56

Chosen confounding factors: sex, age, smoking and alcohol

When analysing the study groups in relation to risk factors, OPG rs2073617 (*p* = 0.034) was more frequent in patients with PI who smoked more than 10 cig/day, and BMP-4 rs17563 (*p* = 0.018) or FGF-3 rs1893047 (*p* = 0.014) in patients with PI and Type II diabetes mellitus (Table 4). We did not find any association between patients with history of periodontal disease and the included SNP.

## Discussion

Peri-implant disease is currently one of the most clinically relevant oral disorders [3]. It is usually diagnosed 2–3 years after the implant surgery and appears in up to 20% of dental implants [20]. In our study, the mean time from implant placement to clinical diagnosis of PI was 4.61 + 2.50 years, similar to other studies (Lindhe et al. 2008; Lang et al. 2011; Derks et al. 2016). As expected, the clinical follow-up time was longer in the case group, which highlights the need for lifelong follow-up of patients with dental implants.

The clinical features of our patients were similar to those of previous studies and the overall Spanish population [21]. Smoking is considered a clear inducer of MBL (Rinke et al. 2011), as it directly inhibits osteoblastic and angiogenic proliferation by nicotine, and indirectly suppresses calcium absorption and the production of PTH, OPG and vitamin D (Sgolastra et al. 2015). In our study we were able to recognise a significant association between the presence of PI and the consumption of more than 10 cigarettes/day (0 = 0.01). However, there are studies where this relationship has not been recognised (Aguirre-Zorzano et al. 2015; Dalago et al. 2017). We think that these differences could be due to the lack of homogeneity in defining tobacco consumer’sand the number of cigarettes they smoke.

In relation to the medical history of the patients, we did not observe an association between peri-implant disease and having a systemic disease. As expected, the most prevalent condition in both study groups was hypertension, given the age of our study population. Diabetes mellitus is a well-knows pathology that may be linked to the development of PI (Ferreira et al. 2006). However, most studies that analyse its involvement do not collect important data such as the patients’ glycaemic level (Taylor and Borgnakke 2008), or do not dissociate it from other cofactors, such as smoking or a history of periodontitis (Daubert et al. 2015). In our study we did not recognise any association with this disease, similar to other authors (Roos-Jansaker et al. 2006; Máximo et al. 2008; Costa et al. 2012; Marrone et al. 2013; Renvert et al. 2014; Derks et al. 2016; Rokn et al. 2017). Like Genco et al. (2013), we believe that further studies are needed to properly assess this possible link. Findings of association between PI and other systemic diseases, such as cardiovascular disease, rheumatoid arthritis, osteoporosis, hypothyroidism, depression or liver disease, are also not yet conclusive (Koldsland et al. 2011; Maximo et al. 2008; Renvert et al. 2014; Wang et al. 2022; Strooker et al. 2022).

History of periodontitis and tobacco consumption are two important risk factors for PI [22]. Patients with history of periodontal disease show higher rates of PD, MBL and an increased risk of PI when not following a maintenance therapy [4, 23]. Furthermore, active periodontitis during dental implant treatment favours PI development [24, 25]. The rigorous maintenance therapy followed by our patients may explain why we did not recognise any link between PI and history of periodontitis, as obtainted by other authors [26, 27]. It is known that tobacco use inhibits osteoblastic and vascular proliferation, suppresses calcium absorption and reduces PTH, OPG and vitamin D production, therefore inducing bone loss [28]. In this regard, heavy tobacco consumption (> 10 cig/day) was strongly associated to our patients with PI, as demonstrated in other studies [2, 25].

At the time of clinical diagnosis, the mean PD of the implants with PI of our case group 5.13 + 1.3 mm, with a mean MBL of 5.55 + 1.1 mm and higher number of implants (56.20%) presenting BOP. These data match previous studies (Karoussis et al. 2003; Ferreira et al. 2006; Máximo et al. 2008; Costa et al. 2012; Marrone et al. 2013; de Araujo Nobre et al. 2015; Dalago et al. 2017; Schwarz et al. 2018). In addition, the lack of significant difference in implant location suggests its potential non-impact on peri-implantitis development.

In recent years, there has been a growing interest in recognizing genetic factors related to the development of PI to mirror the case with periodontal disease [5, 29]. Previously, some researchers have suggested the association of IL-1β (-511) to MBL > 0.5 mm in Chinese population with peri-implant disease [30, 31]. Although we know MBL can be the first sign of PI, the final diagnosis must be confirmed by other clinical parameters (BOP, suppuration, increased PD) [2]. The absence on this relation matches the results obtained by other authors [8, 9, 32], which were also performed with similar and more up-to-date diagnostic criteria of PI.

IL-10 is another inflammatory cytokine that plays an important role in bone remodelling by reducing IL-1 and MMP synthesis, enhancing osteoblastic differentiation and inhibiting osteoclastic action [33]. Neither us nor previous studies have been able to recognise its association with PI [9, 11].

Osteoclastic activity is modulated by CD14 and RANKL (Massey and Flanagan, 1999). CD14 protein regulates the differentiation of monocytes into osteoclasts, while RANKL induces osteoclastogenesis in mature osteoclasts (Sørensen et al. 2007). Work on SNPs to date in Serbian and German populations has not found a link between CD14 (-159) and the development of PI (Rakic et al. 2015; Petkovic-Curcin et al. 2017). Similarly, no association of RANKL (-438) has been observed in Iranian and Brazilian populations (Kadkhodazadeh et al. 2013; Ribeiro et al. 2017; Reis et al. 2020). These results coincide with those obtained in our study in the population of the Basque Country [11, 12, 15, 34–38].

Another protein involved in bone remodelling is LTF, a transferrin glycoprotein that is present in salivary secretion and inflammatory neutrophilic granules (Naot et al. 2005). Under physiological conditions, LTF is a salivary antimicrobial and immunomodulatory defence factor against bacterial infections (including peri-implantopathogens) that also stimulates osteoblastic proliferation and differentiation to produce new bone matrix, while inhibiting osteoclastic action by stimulating the binding of OPG to RANK (Naot et al. 2005). Similar to us, only one group of Brazilian authors has studied the LTF SNP (rs1126477), without having recognised a positive association with the appearance of PI (Doetzer et al. 2015) [11, 12, 15, 34–38].

GBP1 (interferon-induced guanylate-binding protein 1) is a GTPase expressed in T lymphocytes and endothelial cells [39]. This enzyme regulates the maturation of intracellular pathogen-infected autophagosomes and the macrophage cell response to PAMPs [39]. GBP1 is known to play an important role in cell-autonomous immunity against intracellular pathogens (Tietzel et al. 2009; Zhu et al. 2013) and it is also implicated in chronic active Epstein–Barr virus infection and inflammatory response suppression [40]. There are still no studies that have analysed the SNP of GBP1 in relation to PI, except our own. Nonetheless, we believe that, given that many important peri-implantopathogens involved in PI are intracellular, such as *Porphyromonas gingivalis, Tanarella forsythia, Actinomyces. actinomycetemcomitans, Treponema denticola*, etc., a mutation of GBP1 could lead to an ineffective inflammatory response against peri-implant pathogens involved in the IP process.

Bone morphogenetic proteins (BMP) like BRINP3 (BMP/Retinoic Acid Inducible Neural Specific 3), are a set of 15 osteoinductive proteins from the transforming growth factor-beta (TGF-β) superfamily located in osteoprogenitor cells [41]. As in our analysis (*p* = 0.012), similar findings have also been found in Brazilian population [42], which demonstrate that BRINP3 mutation rs1935881 is significantly more common in patients with PI. Since the role of these proteins is to regulate osteoblastic differentiation of pluripotent cells, that is, bone regeneration and remodelling, individuals with this SNP could have a disrupted osseointegration response after dental implant placement, promoting the development of PI.

BRINP3 protein is a bone morphogenetic protein belonging to the TGFβ family, a superfamily of proteins with bone neoformation-inducing and connective capacity (Kawano et al. 2004). BMPs are a set of 15 osteoinductive proteins of osteoprogenitor cells, which regulate osteoblast differentiation during bone remodelling and promote bone regeneration (Anderson et al. 2000). They also stimulate the differentiation of pluripotent cells into different cell lines: adipose tissue, cartilage and bone. Only one group of authors (Casado et al. 2015) has recognised the existence of an association between the presence of BRINP3 rs1935881 and the development of IP in the Brazilian population. In view of our concordant results, we consider that this polymorphism could condition an alteration in the peri-implant osseointegration process that favours the appearance of IP.

Osteoprotegerin (OPG) is an osteoblastic molecule that works as RANK receptor antagonist, inhibiting bone resorption [43]. Different authors [14, 44, 45] have shown a relationship between OPG SNP (-1181) and PI in Chinese, Brazilian and Iranian populations. In our study, this SNP was also more statistically frequent in patients with PI who smoked more than 10 cig/day. It is known that smoker individuals have a lower production and serum levels of OPG than non-smokers probably due to the action of nicotine at the osteoblastic level [46, 47]. This, together with the presence of OPG mutation, could explain the biological plausibility between these two processes and why heavy smokers have a higher risk for PI and higher MBL.

A significant association of BMP-4 rs17563 and FGF-3 rs1893047 to patients with PI and Type II diabetes mellitus was also identified in our analysis. These results are similar to those previously obtained by Coelho et al. [13] in a Brazilian population. BMP/FGF protein axis has been reported as an important element in the osseointegration of dental implants, actively involved in bone regeneration and angiogenesis [48, 49]. It is known that genetic mutations of BMP-4 may be associated with decreased bone density in postmenopausal women and reduced adipocyte glucose uptake, thus inducing insulin resistance [50, 51]. Specifically, BMP4 reduces glucose uptake by adipocytes and has an antagonistic effect on insulin signalling, inducing resistance (Ahrens et al. 1993; Bowers & Lane, 2007; Chattopadhyay et al. 2017). These latter functions would explain the recognised association between the presence of the BMP4 rs17563 SNP in diabetic patients with PI (Perera et al. 2019), as we have been able to recognise in our study.

On the other hand, FGF-3 protein regulates the growth of mature pancreatic islets; so, its modification could potentially lead to alterations in insulin secretion [52]. A poor function of both proteins due to these SNPs might explain the increased risk of PI in patients with Type II diabetes mellitus. Only Coelho et al. (2016) have demonstrated the existence of a significant association between BMP4 and FGF3 SNPs and the development of PI amongst Brazilian patients, but their status in relation to the development of diabetes remains unknown to us. Furthermore, we should point to the small number of patients with diabetes mellitus included in our study, so further studies are needed to reinforce our results.

Considering all this, it is important to note that after performing regression analysis, and similar to all the studies to date on the relationship between SNPs and peri-implant disease with updated diagnostic criteria, none of the SNPs initially associated with the presence of PI (GBP1, BRINP3, OPG, BMP4, FGF3) increased the risk of PI. This forces us to interpret our findings with caution and point to a non-genetic susceptibility nature of PI.

To conclude, the limitations inherent in our study should be considered when interpreting the findings. Among these, the main one is that causality cannot be explained with this type of sample, because of its case-control nature. It is noteworthy that, although we did address many potential confounders, certain residual confounding variables were not taken into account, such dietary habits, use of hormone replacement therapy and levels of inflammatory markers. The omission of these factors may have contributed to result variability, and their exclusion could have affected the generalizability of our results. Despite these limitations, we believe this study provides a valuable contribution to the field.

Our aim in the future is to search for associations between the included SNPs (and/or more) and PI and its risk factors. We are currently working to acknowledge the link between inflammatory and bone metabolism SNPs and patients with PI who have history of periodontal disease, diabetes mellitus and poor plaque control and/or lack of maintenance therapy.

## Conclusions

In summary, the overall genetic features of Basque patients match those found by previous authors. This study shows that individuals with dental implant therapy and peri-implantitis from the Basque Country (Spain) under maintenance therapy program do not have a specific genotype of proinflammatory proteins. Nevertheless, our findings light up the current genetic understanding of peri-implantitis in the sense that, patients with PI could have a particular genotype of GBP1 and BRINP3 proteins, which could favour a modified osseointegration, due to an abnormal immunological response to periodontopathogens. The mutations of OPG, BMP-4 and FGF-3 in patients with PI who are heavy smokers or diabetics could explain why these two conditions are risk factors for peri-implantitis.

Although the current results do not stablish preventive strategies or personalized treatments for patients with PI, individuals with a higher risk for this disease could be genetically assessed. Further genetic susceptibility studies are needed in different populations to assess the true role of the SNPs involved in the pathogenesis of this frequent oral disease, in order to stablish preventive programmes and accurate therapies.

## Data Availability

All data generated or analyzed during this study are included in this published article.

## Abbreviations

BMP: Bone morphogenetic protein
BOP: Bleeding on probing
BRINP3: BMP/Retinoic Acid Inducible Neural Specific 3
CD14: Cluster of differentiation 14
DNA: Deoxyribonucleic acid
FGF: Fibroblastic growth factor
GPB: Interferon-induced guanylate-binding protein
IL: Interleukin
LTF: Lactoferrin
MBL: Marginal bone loss
MMP: Matrix metalloproteinase
OPG: Osteoprotegerin
PTH: Parathyroid hormone
RANKL: Receptor Activator for Nuclear Factor κ B Ligand
RT-PCR: Real time polymerase chain reaction
SNP: Single nucleotide polymorphism
SPT: Supportive program therapy
TGF: Tumor growth factor

## References

[1] Albrektsson T, Isidor F. Consensus report of session IV. In: Lang NP, Karring T, editors. Proceedings of the 1st European Workshop on Periodontology. Chicago: Quintessence; 1994. pp. 365–369.

[2] Schwarz F, Derks J, Monje A, Wang HL (2018). Peri-implantitis. J Clin Periodontol.

[3] Renvert S, Persson GR, Pirih FQ, Camargo PM (2018). Peri-implant health, Peri‐implant mucositis, and peri‐implantitis: case definitions and diagnostic considerations. J Clin Periodontol.

[4] Ferreira SD, Silva GL, Cortelli JR, Costa JE, Costa FO (2006). Prevalence and risk variables for peri-implant disease in Brazilian subjects. J Clin Periodontol.

[5] de Lafuente-Ibáñez I, Setien-Olarra A, Aguirre-Urizar JM, Marichalar-Mendia X (2022). Role of proinflammatory mutations in peri-implantitis: systematic review and meta-analysis. Int J Implant Dent.

[6] Thorisson GA, Smith AV, Krishnan L, Stein LD (2005). The international HapMap project web site. Genome Res.

[7] Landegren U, Nilsson M, Kwok PY (1998). Reading bitsof genetic information: methods for single-nucleotide polymor-phism analysis. Genome Res.

[8] Laine ML, Leonhardt Å, Roos-Jansåker AM, Peña AS, Van Winkelhof AJ, Winkel EG, Renvert S (2006). IL-1RN gene polymorphism is associated with periimplantitis. Clin Oral Implants Res.

[9] Melo RF, Lopes BM, Shibli JA, Marcantonio Junior E, Marcantonio RAC, Galli GMT (2012). Interleukin-1β and interleukin-6 expression and gene polymorphisms in subjects with peri-implant disease. Clin Implants Dent Relat Res.

[10] García-Delaney C, Sánchez-Garcés MA, Figueiredo R, Sánchez-Torres A, Gay-Escoda C (2015). Clinical significance of interleukin-1 genotype in smoking patients as a predictor of peri-implantitis: a case-control study. Med Oral Patol Oral Cir Bucal.

[11] Petkovic-Curcin A, Zeljic K, Cikota-Aleksic B, Dakovic D, Tatic Z, Magic Z (2017). Association of cytokine gene polymorphism with peri-implantitis risk. Int J Oral Maxillofac Implant.

[12] Rakic M, Petkovic-Curcin A, Struillou X, Matic S, Stamatovic N, Vojvodic D (2015). CD14 and TNFα single nucleotide polymorphisms are candidates for genetic biomarkers of peri-implantitis. Clin Oral Inv.

[13] Coelho RB, Gonçalves Junior R, Villas-Boas RDM, Bonato LL, Quinelato V, Pinheiro ADR (2016). Haplotypes in BMP4 and FGF genes increase the risk of peri-implantitis. Braz Dent J.

[14] Zhou J, Zhao Y (2016). Osteoprotegerin Gene (OPG) Polymorphisms Associated with Peri-implantitis susceptibility in a Chinese Han Population. Med Sci Monit.

[15] Ribeiro R, Melo R, Tortamano Neto P, Vajgel A, Souza PR (2017). Polymorphisms of Il-10 (-1082) and RANKL (-438) genes and the failure of Dental implants. Int J Dent.

[16] Del Valle AE, López-Vicente J, Martínez-Conde R, Aguirre-Zorzano LA (2018). Current understanding of genetic polymorphisms as biomarkers for risk of biological complications in implantology. J Clin Exp Dent.

[17] Lang NP, Wilson TG, Corbet EF (2000). Biological complications with dental implants: their prevention, diagnosis and treatment. Clin Oral Implant Res.

[18] Armitage GC (1999). Development of a classification system for periodontal diseases and conditions. Ann Periodontol.

[19] Marichalar-Mendia X, Acha-Sagredo A, Rodriguez-Tojo MJ, Rey-Barja N, Hernando-Rodriguez M, Aguirregaviria (2011). Alcohol-dehydrogenase (ADH1B) Arg48His polymorphism in Basque Country patients with oral and laryngeal cancer: preliminary study. Anticancer Res.

[20] Mombelli A, Müller N, Cionca N (2012). The epidemiology of peri-implantitis. Clin Oral Implant Res.

[21] Rodrigo D, Sanz-Sánchez I, Figuero E, Llodrá JC, Bravo M, Caffesse RG (2018). Prevalence and risk indicators of peri‐implant diseases in Spain. J Clin Periodontol.

[22] Polymeri A, Loos BG, Aronovich S, Steigmann L, Inglehart MR (2022). Risk factors, diagnosis, and treatment of peri-implantitis: a cross‐cultural comparison of US and European periodontists’ considerations. J Periodontol.

[23] Roccuzzo M, Bonino F, Aglietta M, Dalmasso P (2012). Ten-year results of a three arms prospective cohort study on implants in periodontally compromised patients. Part 2: clinical results. Clin Oral Implants Res.

[24] Koldsland OC, Scheie AA, Aass AM (2011). The association between selected risk indicators and severity of peri-implantitis using mixed model analyses. J Clin Periodontol.

[25] de Araujo Nobre M, Mano Azul A, Rocha E, Malo P (2015). Risk factors of peri-implant pathology. Eur J Oral Sci.

[26] Marrone A, Lasserre J, Bercy P, Brecx MC (2013). Prevalence and risk factors for peri-implant disease in Belgian adults. Clin Oral Implant Res.

[27] Canullo L, Tallarico M, Radovanovic S, Delibasic B, Covani U, Rakic M (2016). Distinguishing predictive profiles for patient-based risk assessment and diagnostics of plaque induced, surgically and prosthetically triggered peri‐implantitis. Clin Oral Implants Res.

[28] Sgolastra F, Petrucci A, Severino M, Gatto R, Monaco A (2015). Smoking and the risk of peri-implantitis. A systematic review and meta‐analysis. Clin Oral Implant Res.

[29] Yoshie H, Kobayashi T, Tai H, Galicia JC (2007). The role of genetic polymorphisms in periodontitis. Periodontol 2000.

[30] Lin YH, Huang P, Lu X, Guan DH, Man Y, Wei N, Gong P (2007). The relationship between IL-1 gene polymorphism and marginal bone loss around dental implants. J Oral Maxillofac Surg.

[31] Shimpuku H, Nosaka Y, Kawamura T, Tachi Y, Shinohara M, Ohura K (2003). Genetic polymorphisms of the interleukin-1 gene and early marginal bone loss around endosseous dental implants. Clin Oral Implants Res.

[32] Cardoso JM, Ribeiro AC, Palos C, Proença L, Noronha S, Alves RC (2022). Association between IL-1A and IL-1B gene polymorphisms with peri-implantitis in a Portuguese population—a pilot study. PeerJ.

[33] Zhang Q, Chen B, Yan F, Guo J, Zhu X, Ma S (2014). Interleukin-10 inhibits bone resorption: a potential therapeutic strategy in periodontitis and other bone loss diseases. BioMed Res Int.

[34] Reis MBL, Arid J, Flores EKB, Cruz GV, Marañón-Vásquez GA, Souza LKFD (2020). Association between Genetic Polymorphisms in RANK, RANKL and OPG and Peri-implant diseases in patients from the Amazon Region. Braz Dent J.

[35] Naot D, Grey A, Reid IR, Cornish J (2005). Lactoferrin–a novel bone growth factor. Clin Med Res.

[36] Sørensen MG, Henriksen K, Schaller S, Henriksen DB, Nielsen FC, Dziegiel MH (2007). Characterization of osteoclasts derived from CD14 + monocytes isolated from peripheral blood. J Bone Min Met.

[37] Kadkhodazadeh M, Ebadian AR, Gholami GA, Khosravi A, Tabari ZA (2013). Analysis of RANKL gene polymorphism (rs9533156 and rs2277438) in Iranian patients with chronic periodontitis and periim-plantitis. Arch Oral Biol.

[38] Doetzer AD, Schlipf N, Alvim-Pereira F, Alvim‐Pereira CC, Werneck R, Riess O (2015). Lactotransferrin Gene (LTF) Polymorphisms and Dental Implant loss: a Case‐Control Association Study. Clin Implant Dent Rel Res.

[39] Honkala AT, Tailor D, Malhotra SV (2020). Guanylate-binding protein 1: an emerging target in inflammation and cancer. Front Immunol.

[40] Kim BH, Shenoy AR, Kumar P, Das R, Tiwari S, MacMicking JD (2011). A family of IFN-γ-inducible 65-kD GTPases protects against bacterial infection. Science.

[41] Kawano H, Nakatani T, Mori T, Ueno S, Fukaya M et al. (2004). Identification and characterization of novel developmentally regulated neural-specific proteins, BRINP family. Mol Brain Res. 2004;125(1–2):60–75. 10.1016/j.molbrainres.2004.04.001 15193423

[42] Casado PL, Aguiar DP, Costa LC, Fonseca MA, Vieira TC, Alvim-Pereira CC (2013). Different contribution of BRINP3 gene in chronic periodontitis and peri-implantitis: a cross-sectional study. BMC Oral Health.

[43] Wright HL, McCarthy HS, Middleton J, Marshall MJ (2009). RANK, RANKL and osteoprotegerin in bone biology and disease. Cur Rev Musculoskel Med.

[44] Kadkhodazadeh M, Alizadeh Tabari Z, Ardakani MRT, Ebadian AR, Brook A (2012). Analysis of osteoprotegerin (OPG) gene polymorphism in Iranian patients with chronic periodontitis and peri-implantitis. A cross-sectional study. Eur J Oral Implant.

[45] Xu M, Zhang C, Han Y, Yue Z, Shu C, Hou J. Association between osteoprotegerin rs2073618 polymorphism and peri-implantitis susceptibility: a meta-analysis. BMC Oral Health. 2022;22(1):598. 10.1186/s12903-022-02657-6 10.1186/s12903-022-02657-6 PMC9743567 36503538

[46] Lappin DF, Sherrabeh S, Jenkins WM, Macpherson LM (2007). Effect of smoking on serum RANKL and OPG in sex, age and clinically matched supportive-therapy periodontitis patients. J Clin Periodontol.

[47] Liu D, Xu JK, Figliomeni L, Pavlos NJ, Rogers M, Tan A (2002). Expression of RANKL and OPG mRNA in periodontal disease: possible involvement in bone destruction. Int J Mol Med.

[48] Marie PJ (2003). Fibroblast growth factor signaling controlling osteoblast differentiation. Gene.

[49] Guimarães JM, Guimarães IC, Duarte ME, Vieira ME, Vieira T, Vianna VF (2013). Polymorphisms in BMP4 and FGFR1 gene are associated with fracture non-union. J Orthop Res.

[50] Ramesh BL, Wilson SG, Dick IM, Islam FM, Devine A, Prince RL (2005). Bone mass effects of a BMP4 gene polymorphism in postmenopausal women. Bone.

[51] Bowers RR, Lane MD (2007). A role for bone morphogenetic protein-4 in adipocyte development. Cell Cycle.

[52] Arnaud-Dabernat S, Kritzik M, Kayali AG, Zhang YQ, Liu G, Ungles C (2007). FGFR3 is a negative regulator of the expansion of pancreatic epithelial cells. Diabetes.

